# Supplementary material for: The Expansion of the Hellenic Food Thesaurus; Allergens Labelling and Allergens-Free Claims on Greek Branded Food Products
Source: Nutrients. 2022 Aug 19;14(16):3421. doi: 10.3390/nu14163421 (PMC9416583; doi:10.3390/nu14163421)
Supplement: Supplementary file 1 [file nutrients-14-03421-s001.zip › nutrients-1839147-supplementary.pdf]

Table S1*. Prevalence of the 14 allergens in the ingredient list among the 3,859 products in HelTH per food subcategory.*

Food Subcategories	*Milk* *n* (%)	*Cereals* *n* (%)	*Soybean* *n* (%)	*Eggs* *n* (%)	*Nuts* *n* (%)	*Sesame* *n* (%)	*Mustard* *n* (%)	*Sulphite* *n* (%)	*Fish* *n* (%)	*Celery* *n* (%)	*Peanuts* *n* (%)	*Molluscs* *n* (%)	*Crustaceans* *n* (%)	*Lupin* *n* (%)	*At least 1 allergen* *n* (%)
Crème	40 (100)	0 (0)	2 (5)	0 (0)	0 (0)	0 (0)	0 (0)	0 (0)	0 (0)	0 (0)	0 (0)	0 (0)	0 (0)	0 (0)	40 (100)
Milk	172 (100)	0 (0)	3 (2)	0 (0)	0 (0)	0 (0)	0 (0)	0 (0)	0 (0)	0 (0)	0 (0)	0 (0)	0 (0)	0 (0)	172 (100)
Yogurts	170 (100)	23 (14)	6 (4)	3 (2)	8 (5)	10 (6)	0 (0)	0 (0)	0 (0)	0 (0)	0 (0)	0 (0)	0 (0)	0 (0)	170 (100)
Cheese	213 (100)	3 (1)	0 (0)	1 (0)	0 (0)	0 (0)	0 (0)	1 (0)	1 (0)	1 (0)	0 (0)	0 (0)	0 (0)	0 (0)	213 (100)
Milk imitation products	2 (4)	2 (4)	19 (39)	0 (0)	17 (35)	0 (0)	0 (0)	0 (0)	0 (0)	1 (2)	0 (0)	0 (0)	0 (0)	0 (0)	36 (73)
Frozen dairy desserts	44 (96)	9 (20)	25 (54)	9 (20)	16 (35)	0 (0)	0 (0)	0 (0)	0 (0)	0 (0)	0 (0)	0 (0)	0 (0)	0 (0)	44 (96)
Eggs	0 (0)	0 (0)	0 (0)	35 (100)	0 (0)	0 (0)	0 (0)	0 (0)	0 (0)	0 (0)	0 (0)	0 (0)	0 (0)	0 (0)	35 (100)
Canned meat	33 (41)	4 (5)	22 (28)	0 (0)	0 (0)	0 (0)	8 (10)	1 (1)	0 (0)	7 (9)	5 (6)	0 (0)	0 (0)	0 (0)	48 (60)
Sausage or similar products	13 (37)	2 (6)	11 (31)	0 (0)	0 (0)	0 (0)	4 (11)	0 (0)	0 (0)	1 (3)	1 (3)	0 (0)	0 (0)	0 (0)	18 (58)
Meat dish	11 (52)	17 (81)	8 (38)	4 (19)	0 (0)	0 (0)	2 (10)	0 (0)	0 (0)	3 (14)	1 (5)	0 (0)	0 (0)	0 (0)	13 (62)
Seafood products	1 (1)	8 (11)	0 (0)	0 (0)	0 (0)	0 (0)	1 (1)	1 (1)	68 (91)	3 (4)	0 (0)	8 (11)	1 (1)	0 (0)	75 (100)
Vegetable fat or oil	3 (38)	0 (0)	0 (0)	0 (0)	0 (0)	0 (0)	0 (0)	0 (0)	0 (0)	0 (0)	0 (0)	0 (0)	0 (0)	0 (0)	3 (38)
Margarine or mixed origin fat	21 (54)	0 (0)	1 (3)	0 (0)	0 (0)	0 (0)	0 (0)	0 (0)	0 (0)	0 (0)	0 (0)	0 (0)	0 (0)	0 (0)	21 (54)
Butter or animal fat	34 (100)	0 (0)	0 (0)	0 (0)	0 (0)	0 (0)	0 (0)	0 (0)	0 (0)	0 (0)	0 (0)	0 (0)	0 (0)	0 (0)	34 (100)
Cereal or cereal milling products	11 (22)	51 (100)	12 (24)	7 (14)	0 (0)	0 (0)	0 (0)	0 (0)	0 (0)	10 (20)	0 (0)	0 (0)	0 (0)	0 (0)	51 (100)
Rice or similar product	7 (7)	27 (28)	14 (14)	5 (5)	0 (0)	0 (0)	3 (3)	0 (0)	1 (1)	0 (0)	0 (0)	0 (0)	0 (0)	0 (0)	28 (29)
Pasta or similar product	30 (15)	194 (97)	1 (0)	29 (14)	0 (0)	0 (0)	0 (0)	0 (0)	1 (1)	0 (0)	0 (0)	0 (0)	0 (0)	0 (0)	194 (97)
Breakfast cereals	63 (42)	137 (92)	65 (44)	0 (0)	26 (17)	2 (2)	0 (0)	12 (8)	0 (0)	0 (0)	11 (7)	0 (0)	0 (0)	0 (0)	145 (97)

Bread or similar product	30 (14)	206 (97)	32 (15)	9 (4)	1 (0)	39 (18)	3 (1)	2 (1)	0 (0)	1 (0)	0 (0)	0 (0)	0 (0)	1 (0)	209 (99)
Fine bakery ware	182 (70)	257 (99)	154 (59)	80 (31)	39 (15)	6 (2)	0 (0)	7 (3)	0 (0)	0 (0)	3 (1)	0 (0)	0 (0)	3 (1)	259 (100)
Savoury cereal dish	82 (95)	86 (100)	21 (24)	31 (36)	0 (0)	4 (5)	2 (2)	0 (0)	0 (0)	3 (3)	1 (1)	0 (0)	0 (0)	0 (0)	83 (97)
Nuts	2 (3)	19 (29)	4 (6)	0 (0)	40 (61)	1 (2)	0 (0)	4 (6)	0 (0)	0 (0)	23	0 (0)	0 (0)	0 (0)	61 (92)
Seeds or Kernel	1 (3)	0 (0)	0 (0)	0 (0)	0 (0)	0 (0)	0 (0)	0 (0)	0 (0)	0 (0)	0 (0)	0 (0)	0 (0)	0 (0)	1 (3)
Nuts or seeds products	1 (4)	0 (0)	6 (22)	0 (0)	2 (7)	21 (78)	0 (0)	0 (0)	0 (0)	0 (0)	4 (15)	0 (0)	0 (0)	0 (0)	27 (100)
Vegetables (excluding potato)	4 (2)	1 (1)	0 (0)	0 (0)	0 (0)	0 (0)	1 (1)	4 (2)	0 (0)	0 (0)	0 (0)	0 (0)	0 (0)	0 (0)	11 (6)
Starchy root or potato	2 (10)	0 (0)	0 (0)	0 (0)	0 (0)	0 (0)	0 (0)	5 (24)	0 (0)	0 (0)	0 (0)	0 (0)	0 (0)	0 (0)	7 (33)
Pulse or pulse product	0 (0)	1 (2)	0 (0)	0 (0)	0 (0)	0 (0)	0 (0)	0 (0)	0 (0)	0 (0)	0 (0)	0 (0)	0 (0)	0 (0)	1 (2)
Processed fruit product	0 (0)	0 (0)	0 (0)	0 (0)	0 (0)	0 (0)	0 (0)	15 (35)	0 (0)	0 (0)	0 (0)	0 (0)	0 (0)	0 (0)	15 (35)
Sugar, honey, or syrup	0 (0)	0 (0)	0 (0)	0 (0)	0 (0)	0 (0)	0 (0)	0 (0)	0 (0)	0 (0)	0 (0)	0 (0)	0 (0)	0 (0)	0 (0)
Marmalade	1 (1)	2 (2)	0 (0)	1 (1)	1 (1)	0 (0)	0 (0)	0 (0)	0 (0)	0 (0)	0 (0)	0 (0)	0 (0)	0 (0)	4 (5)
Sugary products (no chocolate)	1 (1)	0 (0)	2 (3)	3 (4)	14 (21)	38 (56)	0 (0)	6 (9)	0 (0)	0 (0)	3 (4)	0 (0)	0 (0)	0 (0)	49 (72)
Chocolate	159 (77)	49 (24)	182 (88)	1 (0)	75 (36)	1 (0)	0 (0)	1 (0)	0 (0)	0 (0)	8 (4)	0 (0)	0 (0)	0 (0)	199 (96)
Juice or nectar	0 (0)	0 (0)	0 (0)	0 (0)	0 (0)	0 (0)	0 (0)	2 (1)	0 (0)	1 (1)	0 (0)	0 (0)	0 (0)	0 (0)	3 (2)
Non-alcoholic beverage	5 (2)	2 (1)	10 (4)	0 (0)	0 (0)	0 (0)	0 (0)	0 (0)	0 (0)	0 (0)	0 (0)	0 (0)	0 (0)	0 (0)	12 (4)
Spices or condiments	60 (21)	64 (23)	33 (12)	43 (15)	7 (2)	2 (1)	60 (21)	23 (8)	3 (1)	25 (9)	1 (0)	1 (0)	0 (0)	0 (0)	189 (67)
Prepared food product	75 (47)	55 (34)	21 (13)	26 (16)	1 (1)	5 (3)	24 (15)	2 (1)	6 (4)	18 (11)	1 (1)	0 (0)	2 (1)	0 (0)	116 (73)
Ready-to-eat meal	19 (50)	25 (66)	11 (29)	15 (39)	3 (8)	2 (5)	12 (32)	17 (45)	5 (13)	8 (21)	1 (3)	1 (3)	2 (5)	0 (0)	35 (92)
Frozen, semi-ready meal	6 (15)	16 (40)	8 (20)	2 (5)	1 (3)	1 (3)	0 (0)	0 (0)	1 (3)	3 (8)	0 (0)	0 (0)	0 (0)	0 (0)	19 (48)
**Total**	**1498 (39)**	**1260 (33)**	**673 (17)**	**304 (8)**	**251 (7)**	**132 (3)**	**120 (3)**	**103 (3)**	**86 (2)**	**85 (2)**	**63 (2)**	**10 (0.3)**	**5 (0.1)**	**4 (0.1)**	**2640 (68)**

Table S2. *Prevalence of the 14 allergens in a precautionary statement among the 3,859 products in HelTH per food subcategory.*

Food Subcategories	*Milk* *n* (%)	*Cereal* *n* (%)	*Soybean* *n* (%)	*Eggs* *n* (%)	*Nuts* *n* (%)	*Sesame* *n* (%)	*Mustard* *n* (%)	*Sulphite* *n* (%)	*Fish* *n* (%)	*Celery* *n* (%)	*Peanu* *n* (%)
Cream	0 (0)	0 (0)	0 (0)	0 (0)	0 (0)	0 (0)	0 (0)	0 (0)	0 (0)	0 (0)	0 (0)
Milk	0 (0)	0 (0)	0 (0)	0 (0)	0 (0)	0 (0)	0 (0)	0 (0)	0 (0)	0 (0)	0 (0)
Yogurts	0 (0)	8 (5)	4 (2)	7 (4)	12 (7)	10 (6)	0 (0)	0 (0)	0 (0)	0 (0)	7 (4)
Cheese	0 (0)	2 (1)	3 (1)	6 (3)	0 (0)	0 (0)	5 (2)	5 (2)	0 (0)	3 (1)	0 (0)
Milk imitation products	0 (0)	0 (0)	1 (2)	0 (0)	6 (12)	0 (0)	0 (0)	0 (0)	0 (0)	0 (0)	0 (0)
Frozen dairy desserts	0 (0)	16 (35)	11 (24)	18 (39)	22 (48)	4 (9)	0 (0)	0 (0)	0 (0)	0 (0)	18 (39
Eggs	0 (0)	0 (0)	0 (0)	0 (0)	0 (0)	0 (0)	0 (0)	0 (0)	0 (0)	0 (0)	0 (0)
Canned meat	20 (25)	12 (15)	34 (43)	29 (36)	42 (53)	1 (1)	47 (59)	27 (34)	0 (0)	45 (56)	6 (8)
Sausage or similar products	8 (23)	4 (11)	7 (20)	13 (37)	11 (31)	0 (0)	13 (37)	10 (29)	0 (0)	16 (46)	4 (11
Meat dish	8 (38)	2 (10)	0 (0)	3 (14)	3 (14)	0 (0)	9 (43)	0 (0)	0 (0)	3 (14)	0 (0)
Seafood products	1 (1)	1 (1)	1 (1)	1 (1)	0 (0)	0 (0)	1 (1)	0 (0)	0 (0)	1 (1)	0 (0)
Vegetable fat or oil	0 (0)	0 (0)	0 (0)	0 (0)	0 (0)	0 (0)	0 (0)	0 (0)	0 (0)	0 (0)	0 (0)
Margarine or mixed origin fat	12 (31)	0 (0)	0 (0)	0 (0)	0 (0)	0 (0)	0 (0)	0 (0)	0 (0)	0 (0)	0 (0)
Butter or animal fat	0 (0)	0 (0)	0 (0)	0 (0)	0 (0)	0 (0)	0 (0)	0 (0)	0 (0)	0 (0)	0 (0)
Cereal or cereal milling products	25 (25)	0 (0)	15 (29)	28 (55)	15 (29)	21 (41)	16 (31)	1 (2)	1 (2)	5 (10)	4 (8)
Rice or similar product	12 (12)	4 (4)	8 (8)	17 (18)	3 (3)	16 (16)	16 (16)	0 (0)	6 (6)	9 (9)	8 (8)
Pasta or similar product	12 (6)	0 (0)	73 (36)	47 (23)	1 (0)	4 (2)	2 (1)	0 (0)	0 (0)	2 (1)	1 (0)
Breakfast cereals	55 (37)	3 (2)	29 (19)	3 (2)	87 (58)	25 (17)	2 (1)	1 (1)	0 (0)	0 (0)	43 (29
Bread or similar product	87 (41)	2 (1)	38 (18)	79 (37)	46 (22)	100 (47)	1 (0)	0 (0)	0 (0)	1 (0)	4 (2)
Fine bakery wares	46 (18)	0 (0)	56 (22)	104 (40)	163 (63)	73 (28)	5 (2)	12 (5)	0 (0)	5 (2)	65 (25
Savoury cereal dish	3 (3)	0 (0)	24 (28)	35 (41)	36 (42)	61 (71)	38 (44)	2 (2)	2 (2)	21 (24)	1 (1)
Nuts	2 (3)	38 (58)	2 (3)	0 (0)	24 (36)	43 (65)	1 (2)	0 (0)	0 (0)	0 (0)	40 (61
Seeds or Kernel	0 (0)	0 (0)	0 (0)	0 (0)	2 (6)	0 (0)	0 (0)	0 (0)	0 (0)	0 (0)	0 (0)
Nuts or seeds products	2 (2)	8 (30)	0 (0)	0 (0)	3 (11)	4 (15)	0 (0)	3 (11)	0 (0)	0 (0)	0 (0)

Vegetables (excluding potato)	0 (0)	0 (0)	0 (0)	0 (0)	1 (1)	0 (0)	0 (0)	2 (1)	0 (0)	3 (2)	0 (0)
Starchy root or potato	8 (38)	6 (29)	0 (0)	3 (14)	0 (0)	0 (0)	0 (0)	0 (0)	0 (0)	3 (14)	0 (0)
Pulse or pulse product	0 (0)	1 (2)	0 (0)	0 (0)	0 (0)	0 (0)	0 (0)	0 (0)	0 (0)	0 (0)	0 (0)
Processed fruit product	0 (0)	4 (9)	0 (0)	0 (0)	4 (9)	2 (5)	0 (0)	1 (2)	0 (0)	0 (0)	4 (9)
Sugar, honey, or syrup	0 (0)	0 (0)	0 (0)	0 (0)	0 (0)	0 (0)	0 (0)	4 (9)	0 (0)	0 (0)	0 (0)
Marmalade	2 (2)	9 (11)	0 (0)	0 (0)	0 (0)	0 (0)	0 (0)	0 (0)	0 (0)	0 (0)	0 (0)
Sugary products (no chocolate)	2 (3)	1 (1)	1 (1)	2 (3)	31 (46)	4 (6)	0 (0)	5 (7)	0 (0)	0 (0)	32 (47
Chocolate	48 (23)	112 (54)	3 (1)	26 (13)	121 (58)	12 (6)	0 (0)	4 (2)	0 (0)	0 (0)	67 (32
Juice or nectar	0 (0)	0 (0)	0 (0)	0 (0)	0 (0)	0 (0)	0 (0)	0 (0)	0 (0)	0 (0)	0 (0)
Non-alcoholic beverage	10 (4)	2 (1)	3 (1)	0 (0)	3 (1)	1 (0)	0 (0)	0 (0)	0 (0)	0 (0)	1 (0)
Spices or condiments	50 (18)	53 (19)	47 (17)	54 (19)	46 (16)	16 (6)	30 (11)	7 (2)	25 (9)	40 (14)	22 (8)
Prepared food product	46 (29)	56 (35)	51 (32)	20 (13)	18 (11)	15 (9)	26 (16)	2 (1)	32 (20)	55 (34)	16 (10
Ready-to-eat meal	7 (18)	6 (16)	11 (29)	7 (18)	6 (16)	10 (26)	11 (29)	6 (16)	14 (37)	24 (63)	6 (16)
Frozen, semi-ready meal	3 (8)	1 (3)	0 (0)	5 (13)	0 (0)	5 (13)	4 (10)	5 (13)	0 (0)	8 (20)	0 (0)
**Total**	469 (12)	351 (9)	422 (11)	507 (13)	706 (18)	427 (11)	227 (6)	97 (3)	80 (2)	244 (6)	349 (9

Table S3. *Prevalence of allergen-free claims per food subcategory.*

Food Subcategory	*Gluten* *n (%)*	*Milk* *n (%)*	*Soy* n (%)	*Sesame* n (%)	*Nuts* n (%)	*Eggs* n (%)	*Mustard* n (%)	*Celery* *n (%)*	*At least one claim* *n (%)*
Cream	2 (5)	0 (0)	0 (0)	0 (0)	0 (0)	0 (0)	0 (0)	0 (0)	2 (5)
Milk	2 (1)	6 (3)	0 (0)	0 (0)	0 (0)	0 (0)	0 (0)	0 (0)	8 (5)
Yogurts	9 (5)	4 (2)	0 (0)	0 (0)	0 (0)	0 (0)	0 (0)	0 (0)	13 (8)
Cheese	5 (2)	2 (1)	0 (0)	0 (0)	0 (0)	0 (0)	0 (0)	0 (0)	7 (3)
Milk imitation products	23 (47)	35 (71)	5 (10)	1 (2)	2 (4)	1 (2)	1 (2)	1 (2)	35 (71)
Frozen dairy desserts	2 (4)	2 (4)	0 (0)	0 (0)	0 (0)	0 (0)	0 (0)	0 (0)	4 (9)
Eggs	0 (0)	0 (0)	0 (0)	0 (0)	0 (0)	0 (0)	0 (0)	0 (0)	0 (0)
Canned meat	39 (49)	3 (4)	0 (0)	0 (0)	0 (0)	0 (0)	0 (0)	0 (0)	39 (49)
Sausage or similar products	13 (37)	0 (0)	0 (0)	0 (0)	0 (0)	0 (0)	0 (0)	0 (0)	13 (37)
Meat dish	0 (0)	0 (0)	0 (0)	0 (0)	0 (0)	0 (0)	0 (0)	0 (0)	0 (0)
Seafood products	1 (1)	0 (0)	0 (0)	0 (0)	0 (0)	0 (0)	0 (0)	0 (0)	1 (1)
Vegetable fat or oil	0 (0)	0 (0)	0 (0)	0 (0)	0 (0)	0 (0)	0 (0)	0 (0)	0 (0)
Margarine or mixed origin fat	0 (0)	0 (0)	0 (0)	0 (0)	0 (0)	0 (0)	0 (0)	0 (0)	0 (0)
Butter or animal fat	0 (0)	1 (3)	0 (0)	0 (0)	0 (0)	0 (0)	0 (0)	0 (0)	1 (3)
Cereal or cereal milling products	0 (0)	0 (0)	0 (0)	0 (0)	0 (0)	0 (0)	0 (0)	0 (0)	0 (0)
Rice or similar product	1 (1)	0 (0)	0 (0)	0 (0)	0 (0)	0 (0)	0 (0)	0 (0)	1 (1)
Pasta or similar product	5 (2)	0 (0)	0 (0)	0 (0)	0 (0)	0 (0)	0 (0)	0 (0)	5 (2)
Breakfast cereals	0 (0)	0 (0)	0 (0)	0 (0)	0 (0)	0 (0)	0 (0)	0 (0)	0 (0)
Bread or similar product	4 (2)	2 (1)	0 (0)	0 (0)	0 (0)	0 (0)	0 (0)	0 (0)	4 (2)
Bakery products	2 (1)	1 (0)	0 (0)	0 (0)	0 (0)	0 (0)	0 (0)	0 (0)	2 (1)
Savoury cereal dish	0 (0)	0 (0)	0 (0)	0 (0)	0 (0)	0 (0)	0 (0)	0 (0)	0 (0)
Nuts	0 (0)	0 (0)	0 (0)	0 (0)	0 (0)	0 (0)	0 (0)	0 (0)	0 (0)
Seeds or Kernel	0 (0)	0 (0)	0 (0)	0 (0)	0 (0)	0 (0)	0 (0)	0 (0)	0 (0)
Nuts or seeds products	4 (15)	0 (0)	0 (0)	0 (0)	0 (0)	0 (0)	0 (0)	0 (0)	4 (15)
Vegetables (excluding potato)	0 (0)	0 (0)	0 (0)	0 (0)	0 (0)	0 (0)	0 (0)	0 (0)	0 (0)
Starchy root or potato	4 (19)	0 (0)	0 (0)	0 (0)	0 (0)	0 (0)	0 (0)	0 (0)	4 (19)
Pulse or pulse product	0 (0)	0 (0)	0 (0)	0 (0)	0 (0)	0 (0)	0 (0)	0 (0)	0 (0)

Processed fruit product	0 (0)	0 (0)	0 (0)	0 (0)	0 (0)	0 (0)	0 (0)	0 (0)	0 (0)
Sugar, honey, or syrup	0 (0)	0 (0)	0 (0)	0 (0)	0 (0)	0 (0)	0 (0)	0 (0)	0 (0)
Marmalade	0 (0)	0 (0)	0 (0)	0 (0)	0 (0)	0 (0)	0 (0)	0 (0)	0 (0)
Sugary products (no chocolate)	8 (12)	0 (0)	0 (0)	0 (0)	0 (0)	0 (0)	0 (0)	0 (0)	8 (12)
Chocolate	17 (8)	1 (0)	0 (0)	0 (0)	0 (0)	0 (0)	0 (0)	0 (0)	18 (9)
Juice or nectar	4 (2)	4 (2)	0 (0)	0 (0)	0 (0)	0 (0)	0 (0)	0 (0)	4 (2)
Non-alcoholic beverage	6 (2)	0 (0)	0 (0)	0 (0)	0 (0)	0 (0)	0 (0)	0 (0)	6 (2)
Spices or condiments	8 (3)	0 (0)	0 (0)	0 (0)	0 (0)	0 (0)	0 (0)	0 (0)	8 (3)
Prepared food product	14 (9)	1 (1)	1 (0.6)	1 (0.6)	0 (0)	0 (0)	0 (0)	0 (0)	14 (9)
Ready-to-eat meal	0 (0)	0 (0)	0 (0)	0 (0)	0 (0)	0 (0)	0 (0)	0 (0)	0 (0)
Frozen, semi-ready meal	5 (13)	0 (0)	0 (0)	0 (0)	0 (0)	0 (0)	0 (0)	0 (0)	5 (13)
**Total**	178 (4.6)	62 (1.6)	6 (0.2)	2 (0.1)	2 (0.1)	2 (0.1)	1 (0.03)	1 (0.03)	206 (5.3)
